# The run for a million continues: is there still space for traditional techniques beyond molecular testing for indeterminate thyroid nodule cytology?

**DOI:** 10.20945/2359-3997000000277

**Published:** 2020-08-06

**Authors:** Carolina Ferraz

**Affiliations:** 1 Departamento de Medicina Faculdade de Ciências Médicas Santa Casa de São Paulo São Paulo SP Brasil Unidade de Doenças da Tireoide, Divisão de Endocrinologia, Departamento de Medicina, Faculdade de Ciências Médicas da Santa Casa de São Paulo, São Paulo, SP, Brasil

Thyroid nodules are increasingly common in our daily lives and the *duo* “thyroid ultrasound (US) & fine needle aspiration biopsy (FNAB)” remains the gold standard for identifying or ruling out malignancy with high accuracy ( 1 ). However, we know that out of these nodules, about 25% has an undefined cytology, being named “indeterminate samples” or, according to the Bethesda’s Classification System, categories III (atypia of undetermined significance – AUS/follicular lesion of undetermined significance – FLUS) and IV (follicular neoplasm – FN/suspicious for follicular neoplasm – SFN) ( 2 ). About 10 years ago, the standard management for these two categories, had been a diagnostic lobectomy ( 3 ). In this way, in order to minimize the number of unnecessary surgeries, the “run for a million” had its start decreed, and many groups started to run after tools that would reduce the number of indeterminate cytology and thus differentiate benign from malignant samples before a surgical procedure becomes really necessary.

The use of frozen section was one of the first solutions to deal with the nodules with indeterminate cytology ( 4 ). The intention was to define intraoperatively if the surgery could be limited to a lobectomy or if total thyroidectomy or even neck dissection was needed. Real-time elastography appeared in the race when the role of some US criteria, through the use of the ACR-TIRADS classification ( 5 ), gained more evidence in the definition of benignity or malignancy of a nodule. Also others diagnostic images as thyroid scintigraphy, in which the capturing nodule could suggest benignity ( 3 ), were suggested as possible candidates. In addition, immunocytochemistry entered this race by using CITED-1, galectin-3, HBME-1, fibronectin-1 and cytokeratin-19 as possible markers ( 6 ). Nevertheless, the molecular markers are the ones which intensified the contest in the beginning of the 90s and which apparently are gaining more and more advantage over the others ( 6 ).

Nowadays, by mentioning indeterminate nodules, the molecular tests are the ones that stand out. Currently, there are 5 commercially available tests in the world, reaching a high diagnostic potential with sensitivity of 89-94% and specificity of 68-85% ( 6 ). Moreover, most of them also provide prognostic information of the nodule, and thus the potential to influence a therapeutic decision. However, the lack of prospective and independent validations of the latest versions of the tests, population-specific validations and, in particular, the high cost seems to be the biggest barrier towards the finish line.

Thus, two articles in this issue of the *Archives of Endocrinology and Metabolism* (AE&M) have aimed at highlighting two approaches that are still controversial in the literature with regard to the differentiation between benign and malignant nodules in indeterminate samples. Luo and cols *.* ( 7 ) and Vuong and cols. ( 8 ) demonstrated that both, the shear wave elastography and the use of intraoperative frozen section, respectively, are still useful and cheaper techniques, and so, brought them back to the run for a million.

Elastography is the evaluation of the tissue elasticity and its rationale is based on the increased stiffness of malignant over benign nodules similar to the hardness on physical examination. The studies about elastography on thyroid nodules are quite controversial. A meta-analysis of eight studies with a total of 639 nodules concluded that elastography has a high sensitivity and specificity in the evaluation of thyroid nodules, which may be used in conjunction or even instead FNAB to select patients with thyroid nodules for surgery ( 9 ). However, results of other studies are more controversial and suggest a more limited diagnostic value since it may depend on the pressure that each operator places on the nodule and on the depth of the nodule localization ( 10 ). A possible solution may be the shear wave elastography (SWE), in which the speed of the waves emitted by the transducer assesses the stiffness of the nodule.

In the present study by Luo and cols. ( 7 ), SWE is used as a criterion for the refinement of US lesions category ACR-TI-RADS 4. This means that before the FNAB is indicated, the nodule would pass through another “sieve”, further narrowing the biopsy indication and, consequently, reducing an invasive diagnostic approach, improving accurate diagnosis and reducing the number of unnecessary FNAB for benign thyroid nodules. An interesting approach that can, consequently, lead to a reduction in the indeterminate samples, through a reproducible technique with little operator interference.

With the increase of preoperative information through cytology, cervical US, tomography scan and even molecular markers, the role of frozen-section analysis of the thyroid has been significantly reduced in recent years. The benefit of frozen biopsy is still very contradictory because its quality depends on the pathologists’ experience in head & neck histology and the size of the tumor. In addition, one of the biggest dilemmas of the indeterminate samples is the follicular lesion because only the access of the entire capsular interface is able to differentiate benign from malignant tumors. So, the identification of capsular and vascular invasion in frozen section analysis can be very difficult and lead to false negative results. However, Vuong and cols. ( 8 ) highlighted the role of intraoperative frozen biopsy, especially in the economic aspect for a possible re-approach of the thyroidectomy. In the present study, completion of thyroidectomy was avoided in 1 out of 24 patients resulting in cost-savings of $80 per patient. Therefore, in places where the access to preoperative information is limited, it seems that the use of intraoperative rapid frozen section may still be an option, due to its affordability.

Collectively, these two papers exemplify that the run for the million has not yet reached the finish line, and more, that each contender runs the way it can: either during initial stages, by helping to better select candidates who really need a more invasive approach, such as FNAB; or with tools that enhance diagnostic surgery. Therefore, it seems that the one million prize will have to be shared, as each technique will prove beneficial depending on the time of the evaluation and the specific country where it is applied, given different reality, accessibility and know-how.
